# The oncology nurse coordinator: role perceptions of staff members and nurse coordinators

**DOI:** 10.1186/s13584-017-0186-8

**Published:** 2017-11-30

**Authors:** Liza Monas, Orly Toren, Beatrice Uziely, David Chinitz

**Affiliations:** 1 Sharett Institute of Oncology, Hadassah-Hebrew University Medical Center, Jerusalem, Israel; 2 Safety and Risk Management Unit, Hadassah-Hebrew University Medical Center, Jerusalem, Israel; 3 Sharett Institute of Oncology, Head Oncology Ambulatory Services Unit, Hadassah-Hebrew University Medical Center, Jerusalem, Israel; 4 Health Policy and Management Braun School of Public Health, Hebrew University-Hadassah Faculty of Medicine, Jerusalem, Israel

## Abstract

**Background:**

There is extensive evidence that the role of nurse coordinators is beneficial for patients. Nurse coordinators are more available to patients compared to general registered nurses, know better to control symptoms and work as team players with multiple care providers. Despite its significance, there is a dearth of literature on the subject in Israel and a lack of clarity regarding the definitions of the role in terms of responsibilities and authorities. The aim of the study is to: To examine how the role of nurse oncology coordinator is implemented in various fields of oncology and to describe the actual performance of different kinds of oncology nurse coordinators and staff perceptions regarding this role in one tertiary hospital in Jerusalem.

**Methods:**

A phenomenological approach was used to explore the participants’ experiences and views of nurse coordinators’ performance. We conducted a qualitative study using in-depth semi-structured interviews. Interviewees included 30 employees from different levels of the hospitals, and leading figures associated with oncology medicine outside of the hospital: Nurses and physicians of the Sharett Oncology Institute of Hadassah Ein Kerem Hospital in Jerusalem, the administrative staff of Hadassah Ein Kerem Hospital, head nurses of the Israel Cancer Association, the chairperson of the Non-Profit Organization of Oncology Nurses, nurse directors at the Ministry of Health Nursing Division, and seven nurse coordinators at Hadassah Ein Kerem Hospital in diverse fields of oncology.

**Results:**

The nurse coordinator is perceived as an important staff member providing care to cancer patients. Several key elements were found to be common features in the work of all nurse coordinators: emotional support, guidance to patients, and coordination of patients’ care.

**Conclusions:**

The nurse coordinator plays a noteworthy role in the health care system. In view of the variety of roles that the nurse coordinator assumes in different units, performance standards must be adapted to the performance areas for each unit, as well as nurses’ professional development requirements. Changes in a service organization and careful attention to the continuum of care highlight the need to develop and to strengthen the role of a nurse who coordinates treatment over the entire continuum of care, both in the hospital and in the community.

**Electronic supplementary material:**

The online version of this article (10.1186/s13584-017-0186-8) contains supplementary material, which is available to authorized users.

## Introduction

Enormous progress has been made in the treatment of cancer, including chemotherapy, radiology, and biological therapies. However, patients and their families, report a lack of sufficient knowledge and information regarding available treatments, healthcare providers, how to communicate with providers, and how to navigate the complicated health care system. Nurse coordinators can play a major role in patients’ encounters with this system [1].

Nurse coordinators were introduced at the Sharett Oncology Institute of the Hadassah Medical Center at Ein Kerem in Jerusalem in the late 1990s. In 2011, seven nurses were working as nurse coordinators at the Institute, where they participated in the treatment of 23,000 patients. Each nurse specializes in a different type of cancer, such as gastrointestinal tumors, melanoma, breast cancer, and others. From the patients’ perspective, cancer treatment coordinators were found to play an important role in inter-staff work on the oncology unit [2]. Nonetheless, a well-defined description of the functions of nurse coordinator and policies regarding the development of this role, and the forms it takes in different oncology subspecialties are lacking. The current study is a qualitative descriptive exploratory study focusing on the actual practices of different kinds of oncology nurse coordinators, and staff perceptions regarding this role.

## Background

Changes in the healthcare system in Israel in the 1990s led to the establishment of new nursing functions, including, during that decade, nurse coordinators in various fields of medicine such as oncology [3]. Patients with malignant diseases frequently experience transitions between acute and outpatient settings during the course of their illness, and undergo treatment with many healthcare providers, a situation which could benefit from coordination of treatment [4]. Care coordinators are found across the continuum of care and serve as advocates for patients and their families, navigating them within the complex health care system (http://www.nursingworld.org/ccexecutivesummary).

Israel has recognized the role of the nurse coordinator as an important one. According to the role definition of the Israeli Ministry of Health (MOH, see Additional file 1), nurse coordinators should accompany patients across the continuum of care, serve as advocates for patients and their families, and help them navigate the healthcare system and provide patients’ education.

### The contribution of the nurse coordinator

Several articles describe the nurse coordinator’s role and state that the nurse coordinator should mediate between all the system elements, to provide each individual customized care by linking the members of the multi-disciplinary staff, providing evidence-based care and safe treatment based on agreed upon protocols [5–7].The nurse coordinator’s role is regarded as beneficial and necessary, especially in the patient’s first steps in the system [8]. Nurse coordinators also improve the quality of care, contribute to the management of the care of patients with breast cancer [9], identify symptomatic deterioration of lung cancer patients [10], improve the psychological well- being of the patient and his or her family and provide knowledge [11–13]. Similar findings have emerged regarding prostate cancer patients under follow-up [14] and in patients who underwent different types of radiotherapy treatments [15, 16]. According to Tarrant et al. [17], nurse coordinators served as a source of information and support in the case of prostate cancer patients and breast cancer patients [18, 19] and in the case of cancer patients in the community [20].

Nurse coordinators are available to patients, and their assistance aids patients in navigating the healthcare system. Furthermore, it appears that nurse coordinators dealing with chronic conditions, such as CVD, stroke, arthritis, and diabetes, incontinency, long-term psychiatric care and cancer, mediate between and integrate the various entities in the healthcare system, especially community-based resources [21–31]. One study, however, shows that care rendered by nurse coordinators made little difference in the quality of treatment received by terminally ill patients [32] where the treatment is typically of a high standard obviating the need for more coordination.

In Israel, the need for care coordination was highlighted in a 1996 study by Sered on women with breast cancer [33]. According to this study, the nurse coordinator role was first formulated in the field of breast cancer to provide a solution for women who felt isolated in the complex oncology system, and these coordinators were followed by nurse coordinators in other fields. The significance of studying this topic, especially the management of care of patients with breast cancer, stemmed from the high incidence of breast cancer in Israel and from the high probability of cure in the early stages of the disease [19].

It is interesting to ask what progress has been made since Sered published her study in 1996 [33] and whether nurse coordinators have provided benefits to the healthcare system. According to a 2011 study by Chinitz and Uzieli [2], 43% of the oncology care providers in Israel, including physicians and nurses, stated that the development of a position of treatment coordinator would enhance the treatment of cancer patients. Despite its significance, there is a dearth of literature on the subject in Israel and a lack of clarity regarding the definitions of the role in terms of responsibilities and authorities. Therefore, a study of the introduction of nurse coordinators in oncology services in Israel offers an opportunity to understand how the role evolves, unexpected pitfalls that may be encountered over time, and interventions that may be required to shore up the nurse coordinator function.

Therefore, in this study we seek to answer the following questions:

What are the roles performed by nurse coordinators in oncology in the Israeli health care system in general and in the Hadassah Ein Kerem Hospital in particular.
2. How do different team members perceive the definition of the role of nurse coordinator and its actual functioning?
What policies should be adopted by the MOH and hospital administrations to strengthen the role of nurse coordinators in oncology?

## Method

The study used a qualitative methodology based on a phenomenological approach to explore participants’ experiences and views, and to describe the “lived experience” of a phenomenon. Semi-structured in-depth interviews were conducted by the researcher (LM) with 26 staff members at Hadassah-Hebrew University Medical Center at Ein Kerem in Jerusalem as well as four leading health care providers and managers in the state of Israel during 2011. Sampling was purposive, aimed at gaining perspectives of relevant actors interfacing with the nurse coordinator role in the hospital. The interviewees from Hadassah were five physicians who had completed residencies in Oncology at Hadassah’s Sharrett Institute, seven nurse coordinators at Hadassah, and 13 additional nurses who filled various positions in the Sharrett Institute, in Hospital Administration. Outside interviews were conducted with the Head of Nursing of the Israel Cancer Association (ICA), and the Head of the Nursing Division of the Israeli Ministry of Health (MOH) (Table 1). It was decided not to include patients in the sample, before gaining in depth understanding of how staff view the nurse coordinator role. All participants agreed to participate on a voluntary basis; no incentive was offered. The anonymity of specific remarks was preserved in the final report.

**Table 1:** Study Participants

Study Participants	Number of Participants
Nurses, Sharett Institute of Oncology, various ranks	9
Physicians, Sharett Institute of Oncology	5
Administration	4
Head nurses, Israel Cancer Association	2
Chair, Association of Oncology Nurses	1
Head of Nursing, Ministry of Health	2
Nurse care coordinators	7

Interviews were based on a questionnaire that was developed for the study (Additional file 2) The questionnaire explored issues such as the role of the nurse coordinator in the oncology healthcare system, the importance of the position, interpersonal relations, coordinators’ perceived efficiency, their availability, their contribution to patients and staff, and job development of nurse coordinators. Demographic details and professional roles were recorded. Interviews were recorded, with permission, for 26 participants. Five interviews were transcribed because participants did not want to be quoted in the final report, or the interview was conducted by phone. Data were collected until saturation of concepts was reached. Participants subsequently confirmed the final transcripts of their interviews.

In the first stage of the analysis of the interviews, interviewees’ statements were divided into meaningful words or sentences, which could be attributed to categories. Each meaningful record included the speaker, the time of data collection, the interview number, and the page and line number in the transcript. In the second stage of the analysis, the research team developed categories based on the materials, and classified the meaningful words and sentences to the categories and subcategories. This was followed by development of headings that best characterized the contents of each category and the internal division of each category into subcategories. At this point, all the categories were reevaluated and it was determined whether new categories should be added, categories should be merged, or items moved from one category to another. Finally the first author (LM) reread all the material to ensure that all relevant material was classified appropriately. After each interview, the researcher noted interim conclusions, and final conclusions for each interview were recorded at a later date.

**Fig. 1:**
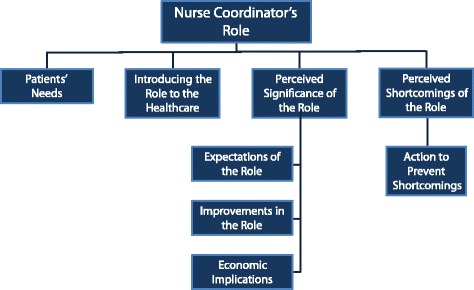
Categorization of Findings

The advanced analysis included: (a) organizing the categories on a vertical axis reflecting their order of importance, and on a horizontal axis to reflect major themes that might be considered supracategories (b) developing a new system of categories and extracting new major topics; (c) developing a hierarchical organization of categories; (d) examining the frequency and prominence of categories (top-down and bottom-up); (e) final conclusions. Finally, we integrated the structural analysis and the thematic analysis. The mapping of the resulting categories and themes is portrayed in Fig. 1.

### Quality assurance of the study

The study observed the usual requirements demanded of qualitative research, including deployment of tools such as peer debriefing, triangulation and recursive analysis, as described in the methodological (Appendix 2).

## Results

### Nurse coordinators’ role

#### Patients’ needs

In 1976, the Israel Cancer Association (ICA) established a group of nurse coordinators**.** Their first assignment was to assist cancer patients with a stoma, provide patient education on stomas, and guide patients on how to navigate the healthcare system.

Based on the success of this first group, additional nurse coordinators were gradually developed in other fields such as palliative care, and community-based nurse coordinators were recruited to assist cancer patients generally, and breast cancer patients specifically. According to several interviewees, breast cancer nurse coordinators are considered one of the greatest successes of the ICA, and their practice model influenced the format of coordination used in other specialties. Nurse coordinators gradually appeared in neuro-oncology and neurosurgery, and later in melanoma, gastrointestinal medicine, neuro-endocrinology, and radiotherapy. Today in Israel there are 30 hospital-based breast cancer nurse coordinators working in oncology.

#### Introducing the role to healthcare system

As hospitals began to recruit nurses as coordinators, the need to define the position emerged, and in the 1990s, the Israeli MOH established this definition. The outline of the role is broad and generic, covering a range of functions and assistance that may vary according to specialty and needs of specific patients and their families (Additional file 1).

### Description of nurse coordinator’s role

#### Principles

Nurse Coordinators maintain multiple ties with multidisciplinary members of the medical and administrative staff, including physicians, staff nurses, social workers, and psychologists, enhancing patients’ sense of trust in the health care system. Some patients need day-to-day contact with someone in a coordinating role. Below are statements by nurse coordinators at Hadassah Hospital:

“…*The nurse is at the center and she coordinates all kinds of different units, whether they are units inside the hospital, the patient, the family, or in the community. ... The major part of the coordination work is the connection between the patient and everything that he or she is supposed to receive from the surroundings … everyone gets a custom-tailored suit.”*

#### The role in an inpatient medical oncology unit

At Hadassah Ein Kerem Medical Center, the position of nurse coordinator was developed in response to the needs of ambulatory oncology patients, who receive services from the different units of the Oncology Institute. Coordination services are rendered by these nurses based on their extensive clinical and management experience within and beyond their personal specializations. Cancer patients are particularly dependent on physicians who are seen as holding the patient’s life in their hands. At the same time, patient’s interactions with physicians are not always intimate.

One physician pointed out that the interactions of ambulatory cancer patients with the clinical staff are extremely brief. The patient knows the physician; however, the brevity of interactions and the lack of intimacy can lead to the former actually being somewhat afraid of the latter. The nurse coordinator is the one constant contact for the patient who sees other members of the staff incidentally during ambulatory visits.

#### Expanding the role

Nurse Coordinators also teach at nursing schools, give lectures to other stakeholders, conduct research, and are engaged in social action. Moreover, they move between the various hospital units according to the needs of their patients and domain of practice. Their position allows them to gain additional knowledge and independence; it also poses professional challenges, and in general develops the field of nursing laterally.

### Perceived significance of nurse coordinator role

#### Economic implications of nurse coordinators

According to the ICA, the nurse coordinator should meet the patient’s needs and the needs of the patient’s family, to ensure that the patient receives the optimal care from the healthcare system with budgetary restrictions.

Participants from the Hadassah Ein Kerem Medical Center, including the Hospital’s Chief Executive, as well as the Israeli Oncological Nursing Society, and the ICA appreciate the financial significance of nurse coordinators.

“*…It also seems to me that if a test gets lost, it is easier to repeat it efficiently* [request by the nurse coordinators] *and not create havoc, and unnecessary costs for the system by going through another visit to the doctor to receive another order for the test, and someone else will do that, to repeat the procedure more. … I have no doubt that it saves the patient a lot of running around and red tape, unnecessary waiting time for unnecessary tests, appointments to see all kinds of doctors,*” states one nursing administrator.

In general, the staff nurses are less involved and less aware of the financial aspects of their role. Nurse coordinators increase the efficiency of outpatient procedures, it is possible that they are indirectly saving the system money, as mentioned above.

A senior nurse at Hadassah’s Sharrett Institute said, “*It’s good that there’s a double explanation for what we do. You need a liaison for the doctor, because doctors can’t answer patients’ phone calls all the time; [the nurse coordinator] can answer some of the questions on her own. If she can’t, then she can ask the doctor on behalf of the patients. I think that’s a huge help. Making appointments is a bit of a problem – she can’t schedule all the appointments…but wherever there is some problem, I mean, either if something is urgent, that’s where they should come in. They need enormous knowledge in the field that they are coordinating, the field they’re in.* …”.

A senior physician agreed, “*Financially, this will bring more patients; patients will get better treatment and better care. They’ll have someone to turn to, they’ll be able to get help in coordinating tests, imaging and other tests. They’ll have one more person to contact and they will feel that they are getting better treatment. It will save time for the physicians and increase the number of patients that come in. …*”.

“*A hospital that wants to be at the center of improved service, meeting the needs of patients and their families, will develop coordination services. The hospital should develop coordination services because it contributes to the cost effectiveness of treatment. It contributes to patients’ cooperation, and coordination between the different systems. Nurse Coordinators will lead to a better flow of treatment,*” stated an administrator at the Nursing Division.

#### Expectations of coordinators’ role

The role should ideally contribute to the nurses’ professional development. The nurse coordinator must become familiar with the relevant population, identify its needs, study the relevant medical issues, and promote treatment. This career horizon will attract more nurses to the profession and help hospitals retain those seeking additional professional development; thus, professional clinical and academic training for nurse coordinators is very important.

It is interesting to note that the interviews did not elicit views regarding the impact of introducing the role of nurse coordinator on healthcare services from respondents representing the Nurses Union or Clalit Healthcare Services. The implications are dealt with in the discussion section.

### Perceived shortcomings of the nurse coordinator role

#### Constraints and shortcomings

As can be seen from Table 2, nurse coordinators play somewhat different roles in the different departments. For example, in surgery, nurse coordinators are involved with much less follow-up as compared with those in the oncology clinic. Since these positions are generally part-time, those playing the role of nurse coordinator devote only a few hours every day to their duties as nurse coordinator. Nurse coordinators thus experience a sense of isolation from one another and from other nurses in the system. The position also has administrative constraints, including budget limitations, uncertainty regarding future institutionalization, and development of the position, and the vagueness of the job description.

**Table 2:** Nurse Coordinators’ Areas of Responsibility at Hadassah Ein Kerem Hospital

Nurse coordinator	Initial meeting with patient	% of full-time position	Key elements of the job	Entity that proposed the position	Funding
Nurse Care Coordinator of breast caner	Surgical and oncology clinic, oncology outpatient clinic, surgery ward	50%	Accompaniment, guidance, consultation, help in coping with the illness, navigation through the system. Additional roles: teaching and research.	Nurse and the Cancer Association	Cancer Association. Second layer of funding by the hospital
Nurse Care Coordinator of skin cancers	Oncology clinic, oncology outpatient clinic	50%	Accompaniment, guidance, consultation, navigation through the system, follow-up, communications with entities in and outside the hospital. Additional roles: teaching.	Nurse and the Cancer Association	Institute foundation, Cancer Association. Second layer of funding by the hospital
Nurse Care Coordinator of gastro-intestinal system cancer	Oncology clinic; oncology outpatient clinic	50%	Accompaniment, guidance, consultation, navigation through the system, follow-up, communications with entities in and outside	Nurse, Physician (head of the field)	Cancer Association, second layer – the Hospital
			the hospital. Additional roles: teaching and research.		
Nurse Care Coordinator of neuro-oncology	Oncology clinic, neuro- surgical clinic, neuro-surgical ward, hematology ward	100%	Accompaniment, guidance, consultation, follow-up, communications with entities in and outside the hospital, facilitation of groups of patients and family members.	Nurse, Physician (head of the field)	Hospital
Nurse Care Coordinator of radiotherapy	Radiotherapy unit	100%	Accompaniment, guidance, consultation, follow-up, communications with entities in and outside the hospital, Direct treatment to patients.	Nurse and Nursing services	Hospital
Nurse Care Coordinator of neuroendocrine tumors	Center for Rare Disease	75%	Accompaniment, guidance, consultation, follow-up, communications with entities in and outside the hospital, Facilitation of patient groups.	Director of the unit	Research fund
Nurse Care Coordinator of neuro-radiosurgery	Radiotherapy unit and neurosurgery ward	60%	Accompaniment, guidance, consultation, follow-up, communications with entities in and outside the hospital, Direct treatment to patients.	Physician and nurse	Hospital

#### Differences between registered nurse and nurse coordinator

Respondents’ view the nurse coordinator, compared to a regular registered nurse, as a nurse who has expanded the boundaries of her profession thanks to the education she received in nursing school. However, while this professionalism provides benefits, there is also risk of a drawback, in the sense that the nurse coordinator may be torn between the hands-on nature of her work as a nurse, and the administrative duties of a coordinator. There may be also tensions between the nurse coordinator and registered nurses, especially as the role is new and not always well established in the organizational structure and culture.

A staff nurse commented, regarding the Nurse Coordinator, “*She is actually a nurse, so sometimes it’s difficult for them and for us to put boundaries on our work. It’s not clear what she has to do and what we have to do. ... Maybe we have overly high expectations of her and that doesn’t always happen. We don’t always know when she has to come into the picture and where we are in it. It’s a position that’s a bit undefined. It’s not clear whether she’s part of the team or not, and where she’s supposed to be, what she’s supposed to do. …*”.

The nurse coordinator’s contribution was sometimes perceived as limited, and they may be considered as quasi-secretaries due to major administrative duties. Several respondents argued that the nurse coordinator should emphasize their own clinical duties, and involve the members of the nursing staff in the patients’ experiences.

Furthermore, nurse coordinators receive limited organizational support. While a substitute must be found for an absent physician or staff nurse, the system does not take care of finding a replacement for an absent nurse coordinator. The system uses other professionals to fill urgent needs that are typically met by the coordinator, and some activities laps. There is a shortage of budgeted positions, and the absence of a formal training program for this position is a critical matter.

#### The reality

The staff is aware of the gap between the existing and desired situation: Under the desired scenario, the nurse coordinator together with nurses on the unit, who admit the patients, all play a very important role in soothing patients, educating them about their care, and improving their access to the system.

Sometimes there is a feeling that the position’s potential is not being realized to its fullest. Furthermore, staff expectations should be aligned and nurse coordinators should be added in fields that do not have any coordinators yet.

## Discussion

Cancer is a common disease with a high mortality rate both in Israel and elsewhere. Due to advances in treatment, many patients are cured; but many of the patients also become chronically ill with a long-term disease, imposing both emotional and physical burdens on them and their families. Complicated illnesses require complicated treatment, and the use of diverse elements of the healthcare system and diverse caregivers requires coordination by nurses within and out of healthcare system [6]. The literature on the subject stresses the need for coordination and provides some analysis of the presence of nurse coordinators on care outcomes. However, the precise ways in which nurse coordinators function and the obstacles confronting stable implementation of the role need to be better understood. This study has contributed important data on these matters.

According to the findings of this study, various staff in a Jerusalem tertiary care hospital consider the role of the outpatient oncology nurse coordinator to be an essential function for the patient, the organization and the physician. In recent years, treatments have increased in the outpatient setting, which explains the significance of nurse coordinators who are accessible and available in various fields of ambulatory oncology. Chinitz and Uzieli [2] found that service providers in the field of oncology in Israel attribute great significance to the development of the nurse coordinator’s role. This strengthens the conclusion that there is a need to refine the definition of the role. This is especially true in the field of oncology which calls for coordination of care across the whole spectrum from diagnosis, through follow up after acute care, to constant monitoring during chronic stages in the community.

One of the main domains associated with the role of the coordinator is ensuring continuity of care: She ponders the patient’s treatment from the medical decision on the type of treatment, accompanies the patient throughout the treatment itself, coordinating the various parties involved in the care cycle. In effect, she serves as a key figure, available, accessible and knowledgeable resource for patients and their families.

Support for this function was found both in studies in the field of oncology and in studies on management of chronic care by nurses [9, 13, 20–31].

The definition of nurse coordinator can rest on the "Strong Model of Advanced Nursing Practice" [34], which includes several domains of practice: coordination, accompaniment, direct care, and patient support. This is supported by findings from this study of nurse coordinators’ practice. A nurse is an excellent liaison person, she has knowledge in a specific field, she is available and able to move between the various hospital units, and knows how to meet the needs of the population that she treats [15, 16, 19]. This is also supported by international definitions of the nurse care coordinator (http://www.nursingworld.org/ccexecutivesummary) and the Nursing Division in MOH (Appendix 1). To these benefits, we can add the finding from the current study that sharper definition of the role of nurse coordinators will have a positive economic effects in terms of more cost effective care.

Compared to this model, our study found little evidence of immediate interface between the hands on care patients receive and the functions of nurse coordinators. The impact of the latter is not readily apparent to the physicians and nurses directly treating patients, making coordination a matter of two steps forward, one step back. Furthermore, an important point that many respondents noted is variability in the performance of different nurse coordinators and the lack of a clear job definition. The differences in performance can be explained by the different requirements of each field of medicine, the diversity of patient needs, the number of patients under each nurse coordinator’s care, the scope of the nurse coordinator’s employment (part- time or full-time), and the nurse coordinator’s personality and role perceptions, as well as the organization that sponsored the introduction of the position in each field of medicine.

At Hadassah Hospital, there is much variation in role performance of nurse coordinators because each nurse coordinator works under different employment conditions (Table 2), leading to overall lack of clarity regarding the nurse coordinator’s job. It is reasonable to assume that such variation exists in other Israeli hospitals as well. Other studies [35, 36] have also found that employees feel that the nurse coordinator’s role in unclear and vague, despite repeated efforts to clarify the role.

This lack of clarity also affects the nurse coordinator’s distribution of labor with the nursing staff, and the relations between the two classes of nurses. Nut and Hungerford [35] emphasize the importance of cooperation between the staff and the nurse coordinator. It should be noted that despite the vagueness and lack of clarity of the role, the oncology unit staff and the nurse coordinators maintain a good relationship, and the staff appreciates the need for nurse coordinators. Nonetheless, some of the staff members who come into contact with nurse coordinators argued that the relationship should be improved in terms of an alignment of expectations of the nurse coordinators and the various staff members who treat the patients directly. In view of the medical developments in the field of oncology, and the developments in the field of nursing, it cannot be denied that the role of the nurse coordinator should be endowed with a modicum of “flexibility”., Nonetheless, clearer definition of the role seems to be the order of the day. Shifting this balance should guide the Nursing Administration in redefining the role and in identifying potential nurse coordinators.

It is also important to build into the role of oncology nurse coordinators communications skills necessary to define the details of their role to other staff members. Our findings indicate that this field of nursing has assumed the responsibility for development of functions that combine organizational knowledge of the healthcare system with clinical knowledge in a specific field, in order to care for the needs of various patient populations. As these responsibilities fall between the cracks of more conventional nursing roles, nurses carrying them out have to be sensitive to the tensions they may face and able to clarify their role to other nurses playing more established roles.

Another interesting impact of the nurse coordinator role that emerges from the findings of this study is that nurse coordinators sometimes prevent hospitalization and unnecessary referrals to the outpatient system. Support for this effect has also been found in the literature, in a study by patients with congestive heart failure, nurse coordinators prevented repeated hospital admissions [20]. It would be useful to assess the magnitude of this impact in Israeli hospitals.

In summary, our findings indicate that the members of the oncology staff, the administration, the MOH and the ICA, view the nurse coordinator as an important and essential professional member of the multi-disciplinary staff. Still, lack of clarity regarding the role and its integration to existing health delivery frameworks remain a challenge.

### Policy issues and implications for policymakers

Our research suggest the following policy options:

### Clear and specific role description of the nurse coordinator role

Despite the significant contribution of this role, and the existing role description of MOH, in reality there is great variation in its actual performance from unit to unit, even within a particular hospital. The current definition is vague, leading to many performance variations with unclear boundaries. A clearer definition should be created by the Ministry of Health and implemented by the health care organizations.

### Maintaining, assessing and developing the role of nurse coordinator

Along with clarification of the nurse coordinator role, specific efforts should be focused on articulating clearly the skills needed for coordination of care among different disciplines inside the hospital, as well as between hospital based and community based care, including ongoing intensive guidance of patients. As the role is performed on the continuum of care, coordination should be examined over the entire continuum, and models for developing the inter-organizational (hospital and community) and organizational (in the hospital) aspects of the role should be examined.

Continual development of the nurse coordinator role requires data on the impact the latter has on performance of health delivery organizations. Our interviews suggest a range of impacts on quality of care, patient experience and cost effectiveness of care. However, as mentioned above, representatives of Clalit Health Services and the Nurses Union did not relate to such impacts, which may indicate that little systematic data is available and will need to be supplied.

### Coordination among staff members

Since the nurse coordinator is the “glue” of a permanent or an alternate staff, it is critically important to develop formal communication among the staff members in order to clarify the boundaries of the different roles. Specific managerial activities should be focused on the formal team as a unit and not merely as individuals working in the same place. These activities will also focus on the team agenda, performance and quality of care, and facilitate measurement of the performance of each member of the team including the nurse coordinator.

### Creating policies and standards at the local level

At the same time, MOH formal definitions should also leave flexibility for each health delivery system to mold its own nurse coordinator model. If the role description is clear and specified, the boundaries of the role and the performance of each nurse coordinator based on local standards will be clearer.

### Nurse coordinator training

There is currently no specific training required of nurse coordinators, neither for entry into the position nor on the job. Because the elements of the job are evolving rapidly, and in view of the research demonstrating the important contribution of this role, it is necessary to delineate the training required and the knowledge nurse coordinators should possess, both when assuming the position and during their tenure. We recommend that nurse coordinators be required to update themselves in a manner similar to the requirements for continuing education in other countries as a condition of retaining certification. Continuing education can be done through participation in conferences, conducting research, and taking part in relevant workshops. Importance attaches to development of the skills of nurse coordinators including: organizational, managerial, ensuring continuity of care, and patient education. Such skill development can be done in-house or through various professional organizations such as the Israel Oncology Nurses Association.

## Conclusions

This study explored the perceptions of nursing professionals, physicians, and patients regarding the role of nurse coordinators in Israeli oncology services. The study was conducted primarily in the context of an Israeli tertiary care hospital, and studied the role perceptions and contributions of the role of nurse coordinator to the various professionals in the organization. The nurse coordinators function as organizational “glue” that brings together all the factors involved in patient care to work synchronously in the patient’s interest, and contribute to organizational and financial efficiencies in patient management. In an era where coordination across the continuum of care has become a central concern, especially for the increasing number of patients with chronic conditions, better understanding of the nurse coordinator role is essential. Indeed, coordination of care has recently been included in Israel’s program of Quality Indicators in the Community Program. Such measurement efforts benefit from knowledge of how the processes aimed at achieving good results in terms such indicators actually play out in the actual delivery of care, and can inform the development of indicators sensitive to condition specific coordination efforts. In the case of oncology, better understanding and clarification of the role of nurse coordinators as provided in this paper constitute important input into such quality improvement efforts and health care management more generally.

### Limitations of the study

This study has several limitations. Previous acquaintance among interviewers and interviewees might have an effect on the interviewees’ answers and willing to expose all of their ideas. The principle investigator is employed in the Sharrett Institute, which could have affected discussion during interviews in some cases.

The study was conducted in one site only – a tertiary care hospital in Jerusalem. While, as mentioned above, findings such as those related to related to within hospital variation in definition of the nurse coordinator role, may be expected in most other Israeli hospitals, other issues raised here may not. For example, boundaries between the nurse coordinator role and that of “regular” nurses may be clearer in other hospitals due to size or management intervention. In a smaller hospital it may be easier for management to clarify and standardize the role of nurse coordinators across many departments. Private hospitals, handling more elective procedures, might not feel the same needs for coordination that would merit development of the nurse coordinator role. Furthermore, the desire of nurses for professional development and advancement may vary according to hospital characteristics such as location, size, and the demands of patients. Still, in a small country like Israel, innovations that take place in any part of the system often become known throughout the health system and tend to spread, so that the findings from this study are likely quite relevant to other settings in Israel.

Furthermore, our sample did not include nurses and other personnel from the four Israeli HMOs, so more data is needed on the role of nurse coordinators across the hospital – community continuum. Other actors, for example from the HMOs, and of course patients, should be studied in the future.

Nonetheless, the triangulation of results among nurses, administrators and MOH officials involved in formulating and implementing the nurse coordinator role strengthen our conviction that most of the findings are transferable to other hospital settings in Israel. At the same time, as mentioned above, we acknowledge the possibility of hospital-specific factors and the importance of future parallel (and comparative) studies in other Israeli hospitals.

As described, while patients were not included in this study, the deeper understanding it has provided will serve as a good basis for investigating patient awareness and attitudes towards the impact of nurse coordinators on the care they receive. In addition, the study sample did not include social workers who work in oncology. While the fact that our respondents did not refer to this function is perhaps a striking finding in and of itself, future research should consider the interface of nurse coordinators with social workers.^1^

### Additional files

Additional file 1: Nurse Coordinator: Ministry of Health Job Description. (DOCX 23 kb)

Additional file 2: Developing and Investigative Items. (DOCX 21 kb)

## Appendix 1: Basic documents relating to nurse coordinators in Israel

Israel Cancer Association website. www.cancer.org.il.

Ministry of Health website. www.health.gov.il. Ministry of Health. Nursing administration circular no. 70. www.health.gov.il/nursing.

www.nursingworld.org/ccexecutivesummary.

www.health.gov.il

## Appendix 2: Steps taken to insure the trustworthiness of this qualitative research

This study employed several criteria that are used to examine the features of a qualitative study:

Reliability The strategies used to ensure reliability in this qualitative study included:

(a) An extended stay in the field. The researcher remained in the field during the interview phase until she felt that she reached a point of saturation concerning the data.

(b) Intensive observations. The researcher observed and collected as much information as possible on all aspects of the interview process, including specific information as well as the atmosphere, participants’ state, facial expressions and body language, as well as other information “between the lines.” Since the majority of the interviews were recorded, the researcher was not required to simultaneously take notes during the interview, which is distracting, and was able to concentrate on these aspects.

(c) Peer review. The study design and interview process were discussed throughout the study with the thesis advisors, a focus group including members of the outpatient care team, and a senior nurse at the Sharett Institute.

(d) Confirmation by the participants. Participants confirmed the contents of their interviews and the initial conclusions; where a participant did not agree with the contents, this was noted, and the contents were used only when triangulated with other confirmed interviews.

This stage certainly contributed to the next stage of writing and reflection.

(e) Data triangulation. Information was cross-checked from several perspectives, including those of the treatment coordinators, medical staff, nursing staff, administrative staff, and other relevant stakeholders.

Feasibility The strategies used to ensure transferability in this study included:

(a) A rich description of the data. The interviews and observations were recorded (26/31 interviews) or transcribed, and notes were taken before, during, and at the conclusion of the interviews (indicating silences, hesitations, interviewees’ body language, the atmosphere, and other information). In addition, the entire process and the researcher’s personal connection to the topic were documented. The researcher’s knowledge and personal worldview were also taken into account and documented during the analysis.

(b) Reflective investigation. At every stage of the study, the decision-making process, research questions and analyses were all reviewed with changes made when appropriate.

(c) Purposeful sampling (discriminate sampling). Sampling was designed to select individuals who would have the best set of professional and personal perspectives and experience to aid contribute insights relevant to the research problem.

Consistency During the entire data processing phase, the researcher returned to the starting point and the interim stages of the research, which enhanced the focus of the writing process, and helped the researcher revise or add relevant data and details.

Coding-recoding: Each interview was coded by the researcher who deliberated on the structure of the categories, their elimination, modification, or addition of new categories to enhance comparisons and correct mistakes. As a result the analysis of the data is based on correct and accurate information.

Neutrality Strategies used to protect the neutrality of information and conclusions included:

(a) Critical reexamination of the analysis and reflection. Critical reexamination was implemented throughout the entire research process, including review of interview transcripts and notes, rich reports, and conclusions. Data and analyses were also reviewed by the study advisor and modifications were incorporated in response to the advisor’s comments.

## Footnotes

1 We thank an anonymous reviewer for drawing our attention to this point.
