# Supplementary material for: The oncology nurse coordinator: role perceptions of staff members and nurse coordinators
Source: Isr J Health Policy Res. 2017 Nov 30;6:66. doi: 10.1186/s13584-017-0186-8 (PMC5707790; doi:10.1186/s13584-017-0186-8)
Supplement: Supplementary file 1 — Nurse Coordinator: Ministry of Health Job Description. (DOCX 23 kb) [file 13584_2017_186_MOESM1_ESM.docx]

**Additional file 1– Nurse Coordinator: Ministry of Health Job Description**

Subject coordinator: 50007260 from 01.01.1900 to 31.12.9999

Date revised: May 9, 2011

Job type: Office

Job category: 40001500 – Hospital nurses

Exempt from tender: No

Ministry: MOH – Former Head Office

Basic rank: 039 Nurses ranks B12 B14.

**Job Description**

Coordinates patient treatments in a unique clinical specialty in the hospital, or coordinates in other specialty fields of nursing such as research, procedures, training, according to instructions of the direct supervisor and head nurse in the hospital.

Coordination of clinical issues:

The nurse coordinator schedules patients’ treatments throughout all stages of preparations, treatment, and release, relating to the field of the coordinator’s specialization; including scheduling with therapists; scheduling tests for patient convenience; conducting follow-up as necessary; and preparing individual training programs for patients and their families, customized

Coordination of professional issues:

• Functions as the professional and logistical manager of all the issues related to the

coordinator’s specialty, coordinating all the functions related to the specialty.

• Organizes group training programs for nurses and others in topics related to the coordinator’s

field of responsibility.

• Attends multi-disciplinary staff meetings on topics in the coordinator’s field of responsibility.

• Maintains contact with entities outside the hospital including professionals and others in the

community, relating to the coordinator’s field of responsibility.

• Is responsible for keeping abreast of professional and managerial knowledge related to the

coordinator’s job.

• Performs additional jobs in the coordinator’s professional field, at the instructions of the coordinator’s direct supervisor.

**Job Requirements**

Licenses

Registered Nurse, accredited by the MOH

Education

Accredited undergraduate degree in nursing

Preferably a degree in nursing

Certificate of registration confirming completion of an accredited training program in a relevant clinical field. In clinical fields where no accredited training program exists, said certificate of registration is not required.

Experience

Three years’ experience in nursing

Languages

Hebrew

Individual attributes

• Good interpersonal relations

• Training skills (for individuals and groups)

• Organizational skills

• Communication skills at all levels

• The ability to use judgment and discretion, the ability to prioritize, based on a broad perspective of the circumstances
