# Supplementary material for: The oncology nurse coordinator: role perceptions of staff members and nurse coordinators
Source: Isr J Health Policy Res. 2017 Nov 30;6:66. doi: 10.1186/s13584-017-0186-8 (PMC5707790; doi:10.1186/s13584-017-0186-8)
Supplement: Supplementary file 2 — Developing and Investigative Items. (DOCX 21 kb) [file 13584_2017_186_MOESM2_ESM.docx]

**Additional file 2: Developing and Investigative Items**

| **Topic** | | **Questions** | **Investigative questions** |
| --- | --- | --- | --- |
| The role of the  nurse coordinator in the oncology healthcare system | 1. What can you say about the nurse  coordinator that you work with?  2. In your opinion, what is the role of the nurse coordinator within the oncology treatment framework? (Positive and negative things about this position?)  3. In your opinion, what is the job definition of the nurse coordinator within the oncology treatment framework? | | How would you improve this  role?  Anything else? |
| Importance of the  position | 4. How is the significance of the nurse  coordinator’s role expressed in your field of  care in oncology?  5. How is the significance of the nurse coordinator’s role for you and for patients expressed? | |  |
| Reasons that the  position was created | 6. (for administrative staff) What are the  reasons that led the establishment of the position of nurse coordinator in the oncology institute (whose initiative was it, what were the reasons, what was the source of funding?)  7. What are the reasons that the position of nurse coordinator was created in your area? | | Anything else? |
| Economic and  social value | 8. Does the position of nurse have economic  value? | | How is this expressed? |

| Interpersonal  relations | 9. What is the nature of your relationship with  the nurse coordinators? | How is this expressed? |
| --- | --- | --- |
| Interviewee’s  background information | 10. Position  11. Tenure in the position  12. How long have you been working with a nurse coordinator?  13. Specialization (chemotherapy, radiotherapy, biological treatment). |  |
